# Data-Driven Approach to Improving the Risk Assessment Process of Medical Failures

**DOI:** 10.3390/ijerph15102069

**Published:** 2018-09-20

**Authors:** Shih-Heng Yu, Emily Chia-Yu Su, Yi-Tui Chen

**Affiliations:** 1 Department of Health Care Management, College of Health Technology, National Taipei University of Nursing and Health Sciences, Taipei 10845, Taiwan; shihhengyu@gmail.com; 2 Graduate Institute of Biomedical Informatics, College of Medical Science and Technology, Taipei Medical University, Taipei 11031, Taiwan; emilysu@tmu.edu.tw; 3 Clinical Big Data Research Center, Taipei Medical University Hospital, Taipei 11031, Taiwan

**Keywords:** failure mode and effects analysis, medical failure, novel data-driven approach, data envelopment analysis, healthcare

## Abstract

In recent decades, many researchers have focused on the issue of medical failures in the healthcare industry. A variety of techniques have been employed to assess the risk of medical failure and to generate strategies to reduce the frequency of medical failures. Considering the limitations of the traditional method—failure mode and effects analysis (FMEA)—for risk assessment and quality improvement, this paper presents two models developed using data envelopment analysis (DEA). One is called the slacks-based measure DEA (SBM-DEA) model, and the other is a novel data-driven approach (NDA) that combines FMEA and DEA. The relative advantages of the three models are compared. In this paper, an infant security case consisting of 16 failure modes at Western Wake Medical Center in Raleigh, North Carolina, U.S., was employed. The results indicate that both SBM-DEA and NDA may improve the discrimination and accuracy of detection compared to the traditional method of FMEA. However, NDA was found to have a relative advantage over SBM-DEA due to its risk assessment capability and precise detection of medical failures.

## 1. Introduction

In recent decades, medical failures, which are referred to as errors or adverse events in a medical service, have attracted much attention in the healthcare industry due to the increasing concern for patient safety [1]. The occurrence of medical failures may result in additional costs and a reduction in medical quality [2]. The U.S. Institute of Medicine reported that preventable medical failures result in 1,000,000 injuries and 44,000–98,000 deaths in hospitalized patients [3] and incur a loss of $17,000,000 [4] each year in America. In Taiwan, approximately 500 medical conflicts each year await legal mediation or trial [5]. Researchers suggest that reducing medical failures is critical for improving patient safety in healthcare systems. The prevention of medical failures may consist of two stages: risk assessment and quality improvement to monitor medical failures that may occur in a system. In general, risk assessment is processed by categorizing medical errors and predicting the probability of their occurrence [6]. The Joint Commission on Accreditation of Healthcare Organizations (JCAHO) requires all accredited hospitals or other healthcare organizations to complete at least one proactive risk assessment annually to assess the risk of errors and to continuously improve quality [7].

The techniques of risk assessment and quality improvement involve a variety of methods to prevent medical failures, including Six Sigma, hazard analysis and critical control points (HACCP), failure mode and effect analysis (FMEA) or healthcare failure mode and effect analysis (HFMEA), the Toyota production system (TPS), hazard and operability studies (HAZOP), total quality management/continuous quality improvement (TQM/CQI), root cause analysis (RCA), and probabilistic risk assessment (PRA) [8]. Among these methods, FMEA is widely accepted and employed to assess the risk of medical failures [9,10,11] and thus serves as a basis for generating preventive actions [12]. The validity of FMEA is commonly noted for the assessment of medical risk by the JCAHO. In FMEA, three indexes, including severity (S), occurrence (O) and detection (D), are applied to assess risk, where S denotes the seriousness of the effect of failure, O is the probability or frequency of the failure, and D represents the probability that the failure will be detected before the impact of the effect is realized. However, weaknesses in the process of assessing risk in FMEA have been reported [13,14]. Based on a review of previous studies, the limitations of FMEA include the following: (1) FMEA may not provide sufficient information regarding S, O, and D because it assumes that the relative importance of S, O and D is equal [13]; (2) due to insufficient discriminative power, the prioritization for the failure mode with different combinations of S, O, and D may yield the same value for the risk priority number (RPN), resulting in difficulty in ranking priorities [15]; and (3) FMEA provides limited corrective information on S, O and D for each failure mode.

Inspired by the technique of data envelopment analysis (DEA), this paper proposes two methods to overcome the weaknesses of FMEA. One is called the slacks-based measure DEA (SBM-DEA) model, and the other is a novel data-driven approach (NDA) that combines FMEA and DEA. The proposed modified DEA models are applied to risk index datasets of S, O and D. The realistic solutions, including complete prioritization and effective mitigation strategy, are generated to provide risk managers in healthcare organizations with insights into the degree of risk of medical errors for each medical service. Furthermore, the proposed models are also helpful for carrying out further process redesign for risk mitigation. 

The subsequent sections of this paper are organized as follows. The methodology is presented in Section 2, in which the basic DEA model is briefly presented and three risk assessment approaches are described. In Section 3, the numerical results of FMEA, SBM-DEA and NDA are presented. Section 4 presents a comparison of the three approaches. Section 5 concludes with a summary of findings.

## 2. Methodology

FMEA was first developed to solve reliability and safety problems in the aerospace industry in the late 1950s. Because FMEA emphasizes the proactive prevention of medical failures rather than solutions, it can assist managers in identifying failures and causes/effects and in eliminating failures by instituting corrective actions in the risk assessment process [16]. In FMEA, a documented method is employed, asking the manager to provide structural and formalized information for the risk control and assessment of potential failures in terms of what might go wrong, what might cause it to go wrong, and what effects it would have [17]. The prioritization of failure modes is determined by the RPN, which is calculated by multiplying the scales of S, O and D. Higher values of RPN imply that corrective action is more urgently needed. The calculation of the RPN is expressed as follows:

RPN = S × O × D
(1)

DEA is a non-parametric analysis technique used to measure the relative efficiencies of decision-making units (DMUs). By using mathematical programming, DEA yields a composite efficiency score between zero and one for each DMU having multiple inputs and outputs. A DMU is said to be efficient if and only if it has an efficiency score of one. In DEA, an assumption of the weights for all of the productive indicators is not required. A set of weights is objectively generated via a programming process in which favorable weights for all DMUs under evaluation are determined by maximizing their efficiency scores. The result obtained from DEA may help decision-makers to identify the inefficient units and to consequently establish improvement strategies.

To overcome the weakness of FMEA, a numerous studies suggest that DEA may be an effective option for enhancing the assessment capability of FMEA [18,19,20]. DEA is a well-known data-driven approach for measuring the relative efficiencies among DMUs [21]. According to the efficiency perspective, DEA aggregates all productive indicators and yields a composite score to reveal the distance between a DMU’s position and efficiency frontier. The frontier is formed by efficient DMUs, also called best -practices, representing the boundary condition for the whole system, which all DMUs can benchmark at their current production technology [22].

In this paper, a slacks-based measure (SBM) was used to assess the risk of failure modes in the healthcare industry. Suppose that there are *n* DMUs, each DMU*_j_* (*j* = 1, …, *n*) uses *m* inputs $x_{ij}$ (*I* = 1, …, *m*) to produce *s* outputs $y_{rj}$ (*r* = 1, …, *s*). Let DMU*_o_* be the DMU under evaluation, and its *i*th input and *r*th output are denoted by $x_{io}$ and $y_{ro}$, respectively. The SBM score of DMU*_o_*, calculated using the input orientation, is expressed by the following programming model:

$$\mathrm{Minimize}\;\theta_{o}^{SBM}=1-\frac{1}{m}\overset{m}{\underset{i=1}{\sum }}s_{i}^{-}/x_{io}$$

s.t.

(2) $$\begin{array}{cc} x_{io}=\overset{n}{\underset{j=1}{\sum }}\lambda_{j}x_{ij}+s_{i}^{-}\; & \;i=1,\ldots ,m\\ y_{ro}\leq \overset{n}{\underset{j=1}{\sum }}\lambda_{j}y_{rj}\; & \;r=1,\ldots ,s\\ \lambda_{j}\geq 0,s_{i}^{-}\geq 0\; & \;j=1,\ldots ,n \end{array}$$

Model (2) is commonly referred to as the envelopment form and is the dual problem of the multiplier form. In model (2), $s^{-}\in R^{m}$ is the slack vector for the input, and $\lambda_{j}\in R^{n}$ is the non-negative vector connecting all inputs and outputs to form an efficiency frontier. $s_{i}^{-}$ denotes the excess amounts in the input that can be decreased non-radially comparing with efficient DMUs. The $\theta_{o}^{SBM*}$ is the SBM efficiency of DMU*_o_*. If $s_{i}^{-*}>0$, DMU*_o_* is identified as an inefficient unit, i.e., $\theta_{o}^{SBM*}<1$; otherwise, it is an efficient unit. Compared with a traditional radial DEA model, SBM provides a clearer view for determining the specific input variable that causes the inefficiency. Through the optimal slack amount, $s_{i}^{-*}$, the direction for improving inefficient DMUs can also be easily obtained.

**Figure 1:**
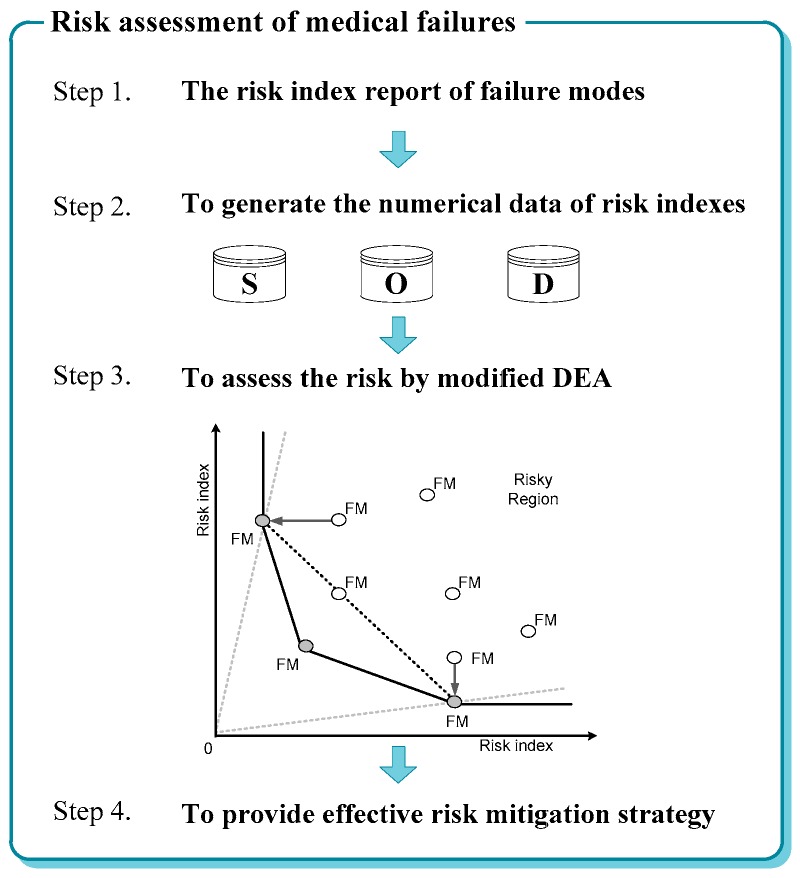
The process of risk assessment. DEA: data envelopment analysis.

Figure 1 depicts the process of risk assessment, including four steps:

Step 1: Collect the risk index report of failure modes.

Step 2: Generate a dataset of risk indexes (severity, occurrence, and detection).

Step 3: Assess the risk of failure modes using modified DEA, including SBM-DEA and NDA, as discussed in this paper, and generate the risk rankings for all failure modes.

Step 4: Provide improvement strategies by mitigating risk for the prevention of medical failures.

Because the S, O, and D dataset in FMEA has the property of “the lower, the better”, which is similar to inputs, this paper considers the risk indexes of S, O, and D as inputs. Thus, by applying SBM, as depicted in Equation (2), the FMEA model yields the following:

$$\mathrm{Minimize}\;\theta_{o}^{RPN}=1-\left(s^{s-}/S_{o}+s^{O-}/O_{o}+s^{D-}/D_{o}\right)/3$$

s.t.

(3) $$\begin{array}{ll} S_{o}=\overset{n}{\underset{j=1}{\sum }}\lambda_{j}S_{j}+s^{S-}\; & \mathrm{for}\;\mathrm{severity}\\ O_{o}=\overset{n}{\underset{j=1}{\sum }}\lambda_{j}O_{j}+s^{O-}\; & \;\mathrm{for}\;\mathrm{occurrence}\\ D_{o}=\overset{n}{\underset{j=1}{\sum }}\lambda_{j}D_{j}+s^{D-}\; & \;\mathrm{for}\;\mathrm{detection}\\ 1=\overset{n}{\underset{j=1}{\sum }}\lambda_{j}\; & j=1,\ldots ,n\\ \lambda_{j},s^{S-},s^{O-},s^{D-}\geq 0 & \end{array}$$

where $s^{S-}$, $s^{O-}$ and $s^{D-}$ denote the slack for S, O, and D, respectively. The $\theta_{o}^{RPN*}$ represents a composed RPN index for each failure mode and is further employed as the basis for prioritizing *n* failure modes. A failure mode with $\theta_{o}^{RPN*}=1$ and $s^{S-*}=0,\;s^{O-*}=0,\;s^{D-*}=0$ represents a safer mode, whereas failure modes with $\theta_{o}^{RPN*}<1$ represent riskier modes. For risky failure modes, the improving targets $\left(S_{o}-s^{S-*},O_{o}-s^{O-*},D_{o}-s^{D-*}\right)$ may be generated automatically by using the optimal slacks ($s^{S-*}$, $s^{O-*}$, $s^{D-*}$). In this paper, Equation (3) is referred to as the SBM-DEA model.

SBM-DEA may successfully improve the discriminatory problem of traditional RPN, i.e., some failure modes have the same RPN composed of different combinations of S, O and D, providing an alternative adjustment as quantitative information for each risky failure mode. However, SBM-DEA may present two chief shortcomings: (1) for safer failure modes, it is unable to generate the complete prioritization, as they all have a unity score $\theta_{o}^{RPN*}=1$; and (2) for risky failure modes, the improving targets waste of risk mitigation resources. 

To overcome the problem arising from SBM-DEA, this paper presents the NDA, expressed in Equations (4) and (5). All failure modes are classified into two sets: safe failure modes and risky failure modes. We then apply the Super SBM model developed by Tone [23] and a minimum distance model developed by Aparicio et al. [24] to evaluate the RPN indexes of safe failure modes and risk failure modes, respectively.

For safer failure modes:

Assume that there are *n* safe failure modes with $\theta_{o}^{RPN*}=1$. To differentiate them, the RPN for each safe failure mode is defined as the optimal value $\delta_{o}^{RPN*}$ in the following model.

$$\mathrm{Minimize}\;\delta_{o}^{RPN}=1+\left(s^{S+}/S_{o}+s^{O+}/O_{o}+s^{D+}/D_{o}\right)/3$$

s.t.

(4) $$\begin{array}{ll} S_{o}\geq \overset{n-1}{\underset{j=1,j\neq o}{\sum }}\lambda_{j}S_{j}-s^{S+}\; & \;\mathrm{for}\;\mathrm{severity}\\ O_{o}\geq \overset{n-1}{\underset{j=1,j\neq o}{\sum }}\lambda_{j}O_{j}-s^{O+} & \mathrm{for}\;\mathrm{occurrence}\\ D_{o}\geq \overset{n-1}{\underset{j=1,j\neq o}{\sum }}\lambda_{j}D_{j}-s^{D-}\; & \mathrm{for}\;\mathrm{detection}\\ 1=\overset{n-1}{\underset{j=1,j\neq o}{\sum }}\lambda_{j}\; & \;j=1,\ldots ,n-1,\;j\neq o\\ \lambda_{j},s^{S+},s^{O+},s^{D+}\;\geq 0 & \end{array}$$

For risky failure modes:

Let *E* be the set of safe failure modes with $\theta_{o}^{RPN}=1$ and $\lambda_{j}=1$. Thus, the RPN index for each risky failure mode can be solved by the following model.

$$\mathrm{Maximize}\;\theta_{o}^{RPN}=1-\left(s^{s-}/S_{o}+s^{O-}/O_{o}+s^{D-}/D_{o}\right)/3$$

s.t.

(5) $$\begin{array}{ll} S_{o}=\overset{n}{\underset{j=1,\;j\in E}{\sum }}\lambda_{j}S_{j}+s^{S-}\; & \mathrm{for}\;\mathrm{severity}\\ O_{o}=\overset{n}{\underset{\;j=1,\;j\in E}{\sum }}\lambda_{j}O_{j}+s^{O-}\; & \;\mathrm{for}\;\mathrm{occurrence}\\ D_{o}=\overset{n}{\underset{j=1,\;j\in E}{\sum }}\lambda_{j}D_{j}+s^{D-}\; & \mathrm{for}\;\mathrm{detection}\\ 1=\overset{n}{\underset{j=1,\;j\in E}{\sum }}\lambda_{j}\; & \;j\in E\\ -\left(v^{S}S_{j}+v^{O}O_{j}+v^{D}D_{j}\right)+u=-d_{j} & \\ v^{S},v^{O},v^{D},\;u\geq 1 & \\ 0\leq d_{j}\leq Mb_{j} & \\ 0\leq \lambda_{j}\leq M\left(1-b_{j}\right) & \\ b_{j}\in \left\{0,1\right\} & \\ s^{S-},s^{O-},s^{D-}\geq 0 & \end{array}$$

where M is a large positive variable, and $b_{j}$ is the binary variable. The $v^{S}$, $v^{O}$ and $v^{D}$ are the weights for severity, occurrence and detection, respectively, which comply with the corresponding constraint to the multiplier form.

Compared to SBM-DEA, in the NDA model, the safe failure modes to be evaluated are removed from the safe frontier ($\lambda_{j},\;j\neq 0).$ In addition, all of the slacks ($s^{S-}$, $s^{O-}$ and $s^{D-}$) in the constraints and objective function are modified from positive to negative. Thus, the RPN for each safe failure mode can be obtained as $\delta_{o}^{RPN*}\geq 1$.

### Data Collection in the Healthcare Industry

In this section, we reuse a case from Western Wake Medical Center in Raleigh, North Carolina, which was first introduced by applying FMEA to mitigate the risk of preventing infant abduction in Reichert [25]. The dataset is tabulated in Table 1 and consists of 16 failure modes, which were identified by a managerial team in the medical service process.

## 3. Results

**Figure 2:**
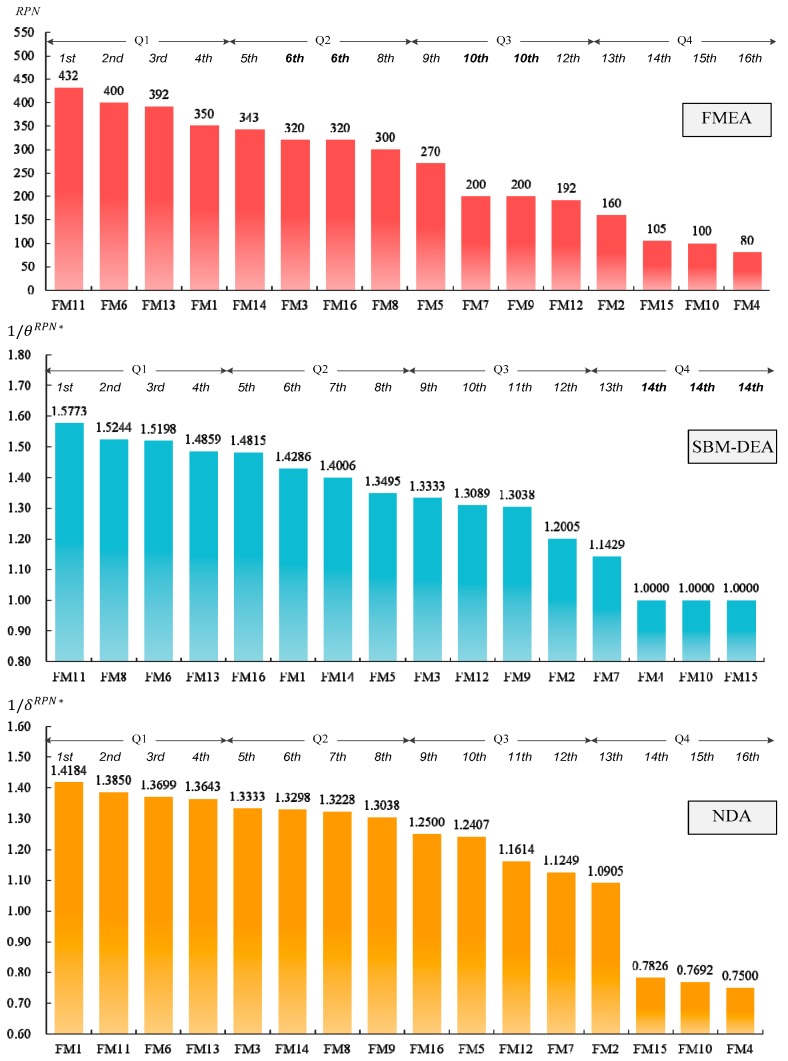
Prioritization of failure modes from risk priority number (RPN), slack-based measure (SBM) and our approach. FMEA: failure mode and effects analysis; SBM-DEA: slacks-based measure; NDA: data-driven approach.

This paper applies the failure mode data in Table 1 to the three models: FMEA, SBM-DEA and NDA. The results are demonstrated in Figure 2 regarding the prioritization of failure modes among the three different approaches. For each approach, the horizontal axis displays the ranked set of failure modes sorted from highest to lowest risk, whereas the risk index is shown on the vertical axis. Additionally, we divided all ranked FMs into four quartiles (designated Q1 to Q4), with Q1 representing the highest 25th percentile of risk for FMs requiring urgent action. In Figure 2, we use the reciprocal of optimal scores obtained by SBM-DEA and the NDA model for simple ranking. A risky FM with $\theta_{o}^{RPN*},\;\delta_{o}^{RPN*}<1$ has the covered risk index $1/\theta_{o}^{RPN*},\;1/\delta_{o}^{RPN*}>1$, which serves as the basis for ranking.

In terms of FMEA, the subsets of the four quartiles included Q1 = {FM 11, FM 6, FM 13, FM 1}, Q2 = {FM 14, FM 3, FM 16, FM 8}, Q3 = {FM 5, FM 7, FM 9, FM 12} and Q4 = {FM 2, FM 15, FM 10, FM 4}. It became clear that the discrimination of FM 3 from FM 16 (an ordinal number of 6th) and FM 7 from FM9 (an ordinal number of 10th) was quite low due to their use of different combinations of S, O and D to compose the same RPN, i.e., FM 3 and FM 16 have RPN = 320; FM 7 and FM 9 have RPN = 200.

SBM-DEA may overcome such discriminatory problems, identifying the following risk priorities: FM 3 ($1/\theta_{3}^{RPN*}=1.333$) ≺ FM 16 ($1/\theta_{16}^{RPN*}=1.482$) and FM 7 ($1/\theta_{7}^{RPN*}=1.143$) ≺ FM 9 ($1/\theta_{9}^{RPN*}=1.304$). However, the ranking is still not sufficiently complete. The risk indexes for FM 4, FM 10 and FM 15 in Q4 imply that they are relative safe modes requiring no corrective action. However, advanced information on the priority of the modes is lacking because these three failure modes have the same unity score. Furthermore, the analytical results for the failure modes in Q1 = {FM 11, FM 8, FM 6, FM 13} and Q2 = {FM 16, FM 1, FM 14, FM 5} by SBM-DEA are not well matched to the results from FMEA. Only 75% of failures in Q1 and 50% in Q2 are in agreement with the FMEA results.

According to the results from the NDA model in Figure 2, all of the problems mentioned above were clearly solved. First, the weak discriminatory power of FMEA was improved upon. Risk rankings between FM 3 and FM 16 and between FM 7 and FM 9 were obtained using the NDA model. The risk index for FM 3 was ($1/\delta_{3}^{RPN*}=1.333$) greater than that for FM 16 ($1/\delta_{16}^{RPN*}=1.25$), and the risk index for FM 7 ($1/\delta_{7}^{RPN*}=1.125$) was smaller than that for FM 9 ($1/\delta_{9}^{RPN*}=1.304$). Second, three safe modes, including FM 4, FM 10 and FM 15, analyzed by SBM-DEA, showed the same unity score. Through the analysis depicted in Equation (4), the risk of these three failure modes was completely ranked using the NDA model. The priority ranking was determined as FM 15 ≻ FM 10 ≻ FM 4. Such a result helps risk managers to obtain full prioritization. Finally, the prioritization of Q1 = {FM 1, FM 11, FM 6, FM 13} and Q2 = {FM 3, FM 13, FM 8, FM 9} based on NDA is virtually a match, with the same results as FMEA, i.e., 100% in Q1 and 75% in Q2.

## 4. Discussion

The RPN value calculated using FMEA provides very limited information for establishing improvement strategies. Compared with the traditional FMEA, both SBM-DEA and the NDA model not only can generate a composite risk index from an efficiency perspective for each FM but also provide the risk control team with the quantitative information to set explicit targets for improving their strategies. This quantitative information can be determined from the difference between risky failure modes and their projection targets. However, these projection targets may differ between SBM-DEA and the NDA model because the former uses the maximum difference, while the latter concerns the minimum difference under programming.

Table 2 provides a detailed list of optimal scores, projection targets and difference rates for all failure modes calculated from SBM-DEA and the NDA model. Both the SBM-DEA model (left-hand side) and the NDA model (right-hand side) identify that FM 4, FM 10 and FM 15 are relatively safe modes. For each, the SBM-DEA model yields a score of one and projection target of S, O and D that are equivalent to the original data, such that the reduction rates are all zero. In contrast, the NDA model provides a score of more than one that can be used as the basis for further ranking. Moreover, the positive rates of S, O and D express additional information on risk-taking ability, i.e., FM 4 can increase 100% of O, FM 10 can increase 40% of O and 50% of D, and FM 15 can increase 83% of S.

**Figure 3:**
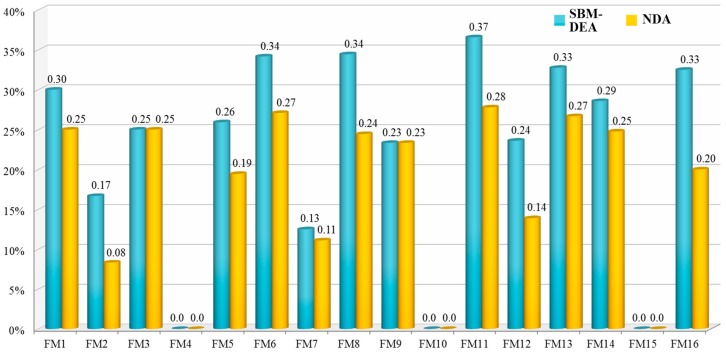
The average reduction rates between SBM and our approach.

For risky failure modes with scores of less than one, the reduction rate with respect to the corresponding original data provides the scales for improving the effort of S, O and D (see the fifth and tenth columns of Table 2). By following these rates, the risky failure modes can be made safer, i.e., either to achieve the projection targets or to produce a score of $\theta_{o}^{RPN*}=1$ for the SBM-DEA model and $\delta_{o}^{RPN*}\geq 1$ for the NDA model. Table 2 also shows large differences in optimal scores and reduction rates between the SBM-DEA model and the NDA model. On the basis of the optimal scores, the first three extreme cases include FM 16 ($\theta_{16}^{RPN*}=0.675$ and $\delta_{16}^{RPN*}=0.800$), FM 8 ($\theta_{8}^{RPN*}=0.756$ and $\delta_{8}^{RPN*}=0.656$) and FM 12 ($\theta_{12}^{RPN*}=0.861$ and $\delta_{8}^{RPN*}=0.764$). Regarding these failure modes, the SBM-DEA model suggests that reduction rates are S (−37.5%), O (−60%), and D (0%) for FM 16; S (−50%), O (−33.33%), and D (−20%) for FM 8; and S (−37.5%), O (−33.33%), and D (0%) for FM 12. These targets seem unattainable. In contrast, the NDA model obviously provides a more attainable (less effort) target for safety improvement with reduction rates of S (0%), O (0%), and D (−60%) for FM 16, S (−33.33%), O (0%), and D (−40%) for FM 8 and S (−16.67%), O (0%), and D (−25%) for FM 12. None of the reduction rates of S, O and D by the NDA model are ensured to be less than those obtained by SBM-DEA. For instance, the NDA model suggests that FM 5 may reach the safety frontier by reducing 8.33% of S and 50% of O, whereas the projection target provided by SBM-DEA asks FM 5 to reduce 44% of O and 33.33% of D. Figure 3 compares the average reduction rates between the SBM-DEA model and the NDA model. The reduction rate calculated by the difference between the projection target and the original data is 21.71% for the NDA model and 22.26% for the SBM-DEA model. The result demonstrates that the projection target of NDA is more closed to FEMA. And thus, on an average, safety improvement recommended by the NDA model are significantly more effective than those provided by the SBM-DEA model.

In brief, SBM-DEA may yield biased measurements of risk and generate unrealistic solutions with two main shortcomings in comparison to the NDA model. First, SBM-DEA imperfectly generates a complete prioritization for all failure modes. Second, the improvement strategy generated by SBM-DEA for risk mitigation is feasible, but it results in a waste of resources.

## 5. Conclusions

Given the growing awareness of and pressure for healthcare quality and patient safety in today’s healthcare environment, both academics and practitioners are increasingly concerned with risk management in medical services to avoid the effects of medical failures. FMEA is a well-known systematic procedure that is widely used to identify medical errors and to provide the necessary corrective actions. In this paper, we presented two models for improving the traditional FMEA method and compared the relative advantages among the three models. The analysis was based on an infant security case at Western Wake Medical Center in Raleigh, North Carolina, U.S.

Both the SBM-DEA model and the NDA model aim to solve the problem of discriminatory power arising from FMEA, which may result in the same RPN with different combinations of S, O and D for some failure modes. The results indicate that the SBM-DEA model and the NDA model, by integrating both two models and FMEA, may provide realistic solutions on optimal targets for safety improvement, whereas the risk mitigation strategies resulting from FMEA remain scarce. However, SBM-DEA might be imperfect, as the corrective direction generated from the optimizing process was unrealistic, resulting in a waste of resources. Additionally, SBM-DEA is incapable of providing complete prioritization for all failure modes.

The NDA model was found to successfully overcome the problems associated with the SBM-DEA model, as the NDA model can provide precise and complete prioritizations of failure modes in the healthcare industry. The NDA model attempts to find the minimum distance for each failure mode and then yields a strategy for improving medical failures. The corrective actions obtained from the NDA model are guaranteed to require less effort. As risk mitigation is a vital activity for reducing any further damages to safety once the risk assessment process is completed, risk mitigation plays a critical role in enhancing safety. The empirical results clarify the superiority of the NDA model. In light of the limited resources of healthcare organizations, the NDA model is a more effective and precise approach for risk management and costs less.

## Figures and Tables

**Table 1:** Risk indexes (S, O, D) in failure mode and effects analysis (FMEA) for preventing infant abduction.

No.	Failure Modes	Severity	Occurrence	Detection
FM1	Child not banded	10	7	5
FM 2	Insufficient IS info provided to mom	5	4	8
FM 3	Mom not paying attention	5	8	8
FM 4	Info not understood	5	2	8
FM 5	Baby may not be HUGS banded prior to washing	10	9	3
FM6	Info not entered into computer system, including name/room	10	8	5
FM7	Delay in entering info into computer system	10	4	5
FM8	“Unfounded” Alarms	10	3	10
FM9	Alarm ringing—doors not locking	10	2	10
FM10	HUGS band not applied until reaching post-partum (sometimes)	10	5	2
FM11	Bands loosening	8	9	6
FM12	Bands not checked and/or tightened properly	8	3	8
FM13	Not checked against census	7	8	7
FM14	Transferred rooms, not updated	7	7	7
FM15	HUGS band may not be checked when moving to nursery, other, for blood draws, circ, etc	5	7	3
FM16	Leaving SCN other than for discharge w/o HUGS band (may include family room visiting)	8	5	8

Note: HUGS: Hugs infant security system SCN: special care nursery.

**Table 2:** Comparative results of SBM-DEA and novel data-driven approach (NDA).

SBM-DEA					NDA				
Modes $\theta_{o}^{RPN*}$	Index	Original	Projection	Rate	Modes $\delta_{o}^{RPN*}$	Index	Original	Projection	Rate
**FM1**	S	10	5.0	−50.00%	**FM1**	S	10	7.5	−25.00%
0.700	O	7	7.0	0.00%	0.705	O	7	3.5	−50.00%
	D	5	3.0	−40.00%		D	5	5.0	0.00%
**FM2**	S	5	5.0	0.00%	**FM2**	S	5	5.0	0.00%
0.833	O	4	2.0	−50.00%	0.917	O	4	4.0	0.00%
	D	8	8.0	0.00%		D	8	6.0	−25.00%
**FM3**	S	5	5.0	0.00%	**FM3**	S	5	5.0	0.00%
0.750	O	8	2.0	−75.00%	0.750	O	8	2.0	−75.00%
	D	8	8.0	0.00%		D	8	8.0	0.00%
**FM4**	S	5	5.0	0.00%	**FM4**	S	5	5.0	0.00%
1.000	O	2	2.0	0.00%	1.333	O	2	4.0	100.00%
	D	8	8.0	0.00%		D	8	8.0	0.00%
**FM5**	S	10	10.0	0.00%	**FM5**	S	10	9.2	−8.33%
0.741	O	9	5.0	−44.44%	0.806	O	9	4.5	−50.00%
	D	3	2.0	−33.33%		D	3	3.0	0.00%
**FM6**	S	10	5.0	−50.00%	**FM6**	S	10	7.5	−25.00%
0.658	O	8	7.0	−12.50%	0.730	O	8	3.5	−56.25%
	D	5	3.0	−40.00%		D	5	5.0	0.00%
**FM7**	S	10	7.5	−25.00%	**FM7**	S	10	6.7	−33.33%
0.875	O	4	3.5	−12.50%	0.889	O	4	4.0	0.00%
	D	5	5.0	0.00%		D	5	5.0	0.00%
**FM8**	S	10	5.0	−50.00%	**FM8**	S	10	6.7	−33.33%
0.656	O	3	2.0	−33.33%	0.756	O	3	3.0	0.00%
	D	10	8.0	−20.00%		D	10	6.0	−40.00%
**FM9**	S	10	5.0	−50.00%	**FM9**	S	10	5.0	−50.00%
0.767	O	2	2.0	0.00%	0.767	O	2	2.0	0.00%
	D	10	8.0	−20.00%		D	10	8.0	−20.00%
**FM10**	S	10	10.0	0.00%	**FM10**	S	10	10.0	0.00%
1.000	O	5	5.0	0.00%	1.300	O	5	7.0	40.00%
	D	2	2.0	0.00%		D	2	3.0	50.00%
**FM11**	S	8	5.0	−37.50%	**FM11**	S	8	6.7	−16.67%
0.634	O	9	7.0	−22.22%	0.722	O	9	3.0	−66.67%
	D	6	3.0	−50.00%		D	6	6.0	0.00%
**FM12**	S	8	5.0	−37.50%	**FM12**	S	8	6.7	−16.67%
0.764	O	3	2.0	−33.33%	0.861	O	3	3.0	0.00%
	D	8	8.0	0.00%		D	8	6.0	−25.00%
**FM13**	S	7	5.0	−28.57%	**FM13**	S	7	7.0	0.00%
0.673	O	8	7.0	−12.50%	0.733	O	8	3.2	−60.00%
	D	7	3.0	−57.14%		D	7	5.6	−20.00%
**FM14**	S	7	5.0	−28.57%	**FM14**	S	7	7.0	0.00%
0.714	O	7	3.0	−57.14%	0.752	O	7	6.2	−11.43%
	D	7	7.0	0.00%		D	7	2.6	−62.86%
**FM15**	S	5	5.0	0.00%	**FM15**	S	5	9.2	83.33%
1.000	O	7	7.0	0.00%	1.278	O	7	7.0	0.00%
	D	3	3.0	0.00%		D	3	3.0	0.00%
**FM16**	S	8	5.0	−37.50%	**FM16**	S	8	8.0	0.00%
0.675	O	5	2.0	−60.00%	0.800	O	5	5.0	0.00%
	D	8	8.0	0.00%		D	8	3.2	−60.00%

Note: FM15 and 16 are taken as two examples to elaborate the calculations of SBM-DEA and NDA (See Appendix A). NDA: novel data-driven approach, SBM-DEA: slacks-based measure DEA (data envelopment analysis).

## Appendix A. Calculations of SBM-DEA and NDA for FM 15 and 16

SBM-DEA solves the following linear problems to obtain the optimal objective values of FM 15 $\theta_{15}^{RPN*}$ = 1.000 and FM 16 $\theta_{16}^{RPN*}$ = 0.675, respectively.

$$\mathrm{Minimize}\;\theta_{15}^{RPN}=1-\left(s^{s-}/5+s^{O-}/7+s^{D-}/3\right)/3$$

s.t.

$$\begin{array}{c} 5=\left(10\lambda_{1}+5\lambda_{2}+5\lambda_{3}+5\lambda_{4}+10\lambda_{5}+10\lambda_{6}+10\lambda_{7}+10\lambda_{8}+10\lambda_{9}+10\lambda_{10}+8\lambda_{11}+8\lambda_{12}+7\lambda_{13}+7\lambda_{14}+5\lambda_{15}+8\lambda_{16}\right)+s^{S-}\;\\ 7=\left(7\lambda_{1}+4\lambda_{2}+8\lambda_{3}+2\lambda_{4}+9\lambda_{5}+8\lambda_{6}+4\lambda_{7}+3\lambda_{8}+2\lambda_{9}+5\lambda_{10}+9\lambda_{11}+3\lambda_{12}+8\lambda_{13}+7\lambda_{14}+7\lambda_{15}+5\lambda_{16}\right)+s^{O-}\;\\ 3=(5\lambda_{1}+8\lambda_{2}+8\lambda_{3}+8\lambda_{4}+3\lambda_{5}+5\lambda_{6}+5\lambda_{7}+10\lambda_{8}+10\lambda_{9}+2\lambda_{10}+6\lambda_{11}+8\lambda_{12}+7\lambda_{13}+7\lambda_{14}+3\lambda_{15}+8\lambda_{16})+s^{D-}\;\\ 1=\left(\lambda_{1}+\lambda_{2}+\lambda_{3}+\lambda_{4}+\lambda_{5}+\lambda_{6}+\lambda_{7}+\lambda_{8}+\lambda_{9}+\lambda_{10}+\lambda_{11}+\lambda_{12}+\lambda_{13}+7\lambda_{14}+\lambda_{15}+\lambda_{16}\right)\;\\ \;\lambda_{1:16},s^{S-},s^{O-},s^{D-}\;\geq 0\; \end{array}$$

$$\mathrm{Minimize}\;\theta_{16}^{RPN}=1-\left(s^{s-}/8+s^{O-}/5+s^{D-}/8\right)/3$$

s.t.

$$\begin{array}{c} 8=\left(10\lambda_{1}+5\lambda_{2}+5\lambda_{3}+5\lambda_{4}+10\lambda_{5}+10\lambda_{6}+10\lambda_{7}+10\lambda_{8}+10\lambda_{9}+10\lambda_{10}+8\lambda_{11}+8\lambda_{12}+7\lambda_{13}+7\lambda_{14}+5\lambda_{15}+8\lambda_{16}\right)+s^{S-}\;\\ 5=\left(7\lambda_{1}+4\lambda_{2}+8\lambda_{3}+2\lambda_{4}+9\lambda_{5}+8\lambda_{6}+4\lambda_{7}+3\lambda_{8}+2\lambda_{9}+5\lambda_{10}+9\lambda_{11}+3\lambda_{12}+8\lambda_{13}+7\lambda_{14}+7\lambda_{15}+5\lambda_{16}\right)+s^{O-}\;\\ 8=(5\lambda_{1}+8\lambda_{2}+8\lambda_{3}+8\lambda_{4}+3\lambda_{5}+5\lambda_{6}+5\lambda_{7}+10\lambda_{8}+10\lambda_{9}+2\lambda_{10}+6\lambda_{11}+8\lambda_{12}+7\lambda_{13}+7\lambda_{14}+3\lambda_{15}+8\lambda_{16})+s^{D-}\;\\ 1=\left(\lambda_{1}+\lambda_{2}+\lambda_{3}+\lambda_{4}+\lambda_{5}+\lambda_{6}+\lambda_{7}+\lambda_{8}+\lambda_{9}+\lambda_{10}+\lambda_{11}+\lambda_{12}+\lambda_{13}+7\lambda_{14}+\lambda_{15}+\lambda_{16}\right)\;\\ \lambda_{1:16},s^{S-},s^{O-},s^{D-}\;\geq 0\; \end{array}$$

NDA solves the following linear problems to obtain the optimal objective values of FM 15 $\delta_{15}^{RPN*}$ = 1.278 and FM 16 $\delta_{16}^{RPN*}$ = 0.800, respectively.

$$\mathrm{Minimize}\;\theta_{15}^{RPN}=1+\left(s^{s+}/5+s^{O+}/7+s^{D+}/3\right)/3$$

s.t.

$$\begin{array}{c} 5\geq \left(10\lambda_{1}+5\lambda_{2}+5\lambda_{3}+5\lambda_{4}+10\lambda_{5}+10\lambda_{6}+10\lambda_{7}+10\lambda_{8}+10\lambda_{9}+10\lambda_{10}+8\lambda_{11}+8\lambda_{12}+7\lambda_{13}+7\lambda_{14}+8\lambda_{16}\right)+s^{S+}\\ 7\geq \left(7\lambda_{1}+4\lambda_{2}+8\lambda_{3}+2\lambda_{4}+9\lambda_{5}+8\lambda_{6}+4\lambda_{7}+3\lambda_{8}+2\lambda_{9}+5\lambda_{10}+9\lambda_{11}+3\lambda_{12}+8\lambda_{13}+7\lambda_{14}+5\lambda_{16}\right)+s^{O+}\\ 3\geq (5\lambda_{1}+8\lambda_{2}+8\lambda_{3}+8\lambda_{4}+3\lambda_{5}+5\lambda_{6}+5\lambda_{7}+10\lambda_{8}+10\lambda_{9}+2\lambda_{10}+6\lambda_{11}+8\lambda_{12}+7\lambda_{13}+7\lambda_{14}+8\lambda_{16})+s^{D+}\\ 1=\left(\lambda_{1}+\lambda_{2}+\lambda_{3}+\lambda_{4}+\lambda_{5}+\lambda_{6}+\lambda_{7}+\lambda_{8}+\lambda_{9}+\lambda_{10}+\lambda_{11}+\lambda_{12}+\lambda_{13}+7\lambda_{14}+\lambda_{16}\right)\\ \;\lambda_{1:16\left(\neq 15\right)},s^{S+},s^{O+},s^{D+}\;\geq 0 \end{array}$$

$$\mathrm{Maximize}\;\theta_{16}^{RPN}=1-\left(s^{s-}/8+s^{O-}/5+s^{D-}/8\right)/3$$

s.t.

$$\begin{array}{c} 8=\left(5\lambda_{4}+10\lambda_{10}+5\lambda_{15}\right)+s^{S-}\\ 5=\left(2\lambda_{4}+5\lambda_{10}+7\lambda_{15}\right)+s^{O-}\\ 8=\left(8\lambda_{4}+2\lambda_{10}+3\lambda_{15}\right)+s^{D-}\\ 1=\left(\lambda_{4}+\lambda_{10}+\lambda_{15}\right)\\ -\left(v^{S}5+v^{O}2+v^{D}8\right)+u=-d_{4}\\ -\left(v^{S}10+v^{O}5+v^{D}2\right)+u=-d_{10}\\ -\left(v^{S}5+v^{O}7+v^{D}3\right)+u=-d_{15}\\ 0\leq d_{4}\leq Mb_{4}\;\;\\ 0\leq d_{10}\leq Mb_{10}\\ 0\leq d_{15}\leq Mb_{15}\\ 0\leq \lambda_{4}\leq M\left(1-b_{4}\right)\\ 0\leq \lambda_{10}\leq M\left(1-b_{10}\right)\\ 0\leq \lambda_{15}\leq M\left(1-b_{15}\right)\\ v^{S},v^{O},v^{D},\;u\geq 1\\ s^{S-},s^{O-},s^{D-}\geq 0\\ \;b_{4},b_{10},b_{15}\in \left\{0,1\right\} \end{array}$$
